# Predictors of delay in the cervical cancer care cascade in Kampala, Uganda

**DOI:** 10.1186/s12905-026-04292-7

**Published:** 2026-02-13

**Authors:** Megan Swanson, Alison El Ayadi, Miriam Nakalembe, Jane Namugga, Carol Nakisige, Lee-may Chen, Megan J. Huchko

**Affiliations:** 1 Department of Obstetrics and Gynecology and Reproductive Sciences, Division of Gynecologic Oncology, University of California, San Francisco, CA USA; 2 Department of Obstetrics, Gynecology and Reproductive Sciences, Bixby Center for Global Reproductive Health, University of California, San Francisco, CA USA; 3 Department of Obstetrics and Gynaecology, Infectious Disease Institute, Makerere University College for Health Sciences School of Medicine, Kampala, Uganda; 4 Department of Obstetrics and Gynaecology, Division of Gynaecologic Oncology, Mulago Specialised Women and Neonatal Hospital, Kampala, Uganda; 5 Division of Gynaecologic Oncology, Uganda Cancer Institute, Kampala, Uganda; 6 Department of Obstetrics and Gynecology, Center for Global Reproductive Health, Global Health Institute, Duke University, Durham, NC USA

**Keywords:** Cervical cancer, Delay, Treatment access, Resource-limited settings

## Abstract

**Background:**

Cervical cancer is the fourth most common cancer among women with significant global disparities in disease burden. In lower-resource settings, where routine screening for cervical cancer is uncommon, higher incidence of advanced-stage disease contributes to increased morbidity and mortality. Understanding care delays may inform strategies to decrease overall time to treatment, and could potentially improve outcomes. We sought to characterize the cervical cancer care cascade and identify factors associated with time to care cascade completion within a Ugandan cohort.

**Methods:**

We collected sociodemographic, reproductive health and care journey data from 268 Ugandan women newly diagnosed with cervical cancer at Mulago National Referral Hospital and the Uganda Cancer Institute. We characterized time from symptoms to presentation (patient interval), time from presentation to diagnosis (diagnostic interval) and time from diagnosis to treatment (treatment interval) and estimated the influence of patient, health provider, system, and disease factors on length of each interval using survival analysis.

**Results:**

Median patient, diagnostic and treatment intervals were 74 days (IQR 26–238), 83 days (IQR 34–229), and 34 days (IQR 18–58), respectively. Patient interval was prolonged by the belief that symptoms would resolve spontaneously (aHR 0.37, 95% CI 0.24–0.57), confusion about where to seek care (aHR 0.64, 95% CI 0.47–0.88), and utilization of traditional care (aHR 0.70, 95% CI 0.51–0.96). Patient interval facilitators included perceiving symptoms as serious (aHR 2.14, 95% CI 1.43–3.19) and suspecting cancer (aHR 1.82, 95% CI 1.12–2.97). Diagnostic interval delays included symptomatic bleeding (aHR 055, 95% CI 0.35–0.85) and visiting > 2 clinics (aHR 0.69, 95% CI 0.49–0.97); facilitators included early-stage disease (aHR 1.41, 95% CI 1.03–1.95) and direct tertiary care presentation (aHR 2.13, 95% CI 1.20–3.79). Treatment interval delays included anticipating long waits (aHR 0.68, 95% CI 0.46–1.02) and requiring blood transfusions (aHR 0.63, 95% CI 0.37–1.07); no facilitators were identified.

**Conclusions:**

We identified potentially modifiable barriers and facilitators along the cervical cancer care cascade. Interventions targeting these factors may reduce delays, but are unlikely to significantly improve morbidity or mortality given advanced-stage disease at symptom acknowledgement and relatively timely treatment initiation. Expanding cervical cancer screening and vaccination are of utmost importance.

## Background

Cervical cancer is the fourth most common cancer among women worldwide, but there is great disparity in disease distribution globally [1, 2]. In Uganda, like many countries in East Africa, cervical cancer is the most common malignancy and is responsible for the greatest cancer-related mortality among women [3–5]. Most women present with advanced-stage disease and 5-year overall survival regionally for all cervical cancer patients is estimated to be 36% [6]. While surgery, chemotherapy and radiation are all services offered at the national public tertiary care facilities, namely Mulago National Referral Hospital (MNRH) and the Uganda Cancer Institute (UCI), multisystem obstacles challenge access to the various treatment modalities [7, 8].

Screening can detect asymptomatic cervical precancerous lesions as well as cancer, allowing for effective preventive treatments or diagnosis at an early stage. Women with invasive cervical cancer may present with symptoms such as bleeding, foul smelling vaginal discharge or pelvic pain. Symptoms are more common in advanced disease. In low- and middle-income countries (LMICs), cervical cancer is rarely diagnosed when asymptomatic as routine effective screening is not readily accessible.

Many women with cervical cancer around the world experience some delay in presentation to care as well as in accurate diagnosis with staging and initiating appropriate treatment [9–14]. Younger age [9, 10, 14] and rural residence [9, 10] are commonly associated with delay in accessing cervical cancer care. However, no single accepted definition of “delay” exists, and women may experience a subjective or objective delay in initial presentation to a clinician, obtaining diagnostic tests, receiving a diagnosis, and/or in initiating treatment (curative or palliative). The United States Centers for Disease Control and Prevention’s National Breast and Cervical Cancer Early Detection Program (NBCCEDP) has set a quality standard of ≤ 90 days from abnormal screening to final diagnosis [15], though this standard may not apply outside of high-income countries with ready access to screening, diagnostic and treatment modalities. There is a paucity of data evaluating the impact of delayed definitive treatment for cervical cancer on survival, especially in advanced disease, particularly in LMICs [16]. However, data from high-income countries suggest that an interval greater than three months from diagnosis to treatment initiation may increase morbidity and decrease survival, even when controlling for stage, age and co-morbidities [17, 18].

“Delay” is not necessarily synonymous with advanced disease. Patients with cervical cancer at any stage may experience delay in accessing care, obtaining a diagnosis and/or initiating treatment. In fact, studies in Uganda and Brazil found that a diagnosis to treatment interval greater than or equal to three months was common overall (55 to 93%, respectively), and that women with advanced cervical cancer were not significantly more likely than those with earlier-stage disease to experience such delay [19, 20].

Reasons for delay in accessing cervical cancer care are speculative and likely vary across the care cascade. Awareness of cervical cancer is generally high; however, misattribution of symptoms and misconceptions regarding etiology are common in Uganda [21–23], and could theoretically contribute to patient delays in seeking medical care. Once care is established, fees associated with labs, imaging and pathology studies can be cost-prohibitive for many families and could result in a delay in obtaining a diagnosis. For instance, one CT scan costs at least as much as the average monthly income nationally (300,000 Ugandan Shillings, about $80 USD). While most medical care, including some basic diagnostic testing has been offered free of charge at public facilities in Uganda since 2001, nearly half of the country’s healthcare financing continues to come from out-of-pocket expenditures [24]. Once a diagnosis has been made, lack of available subspecialists [25], anesthesiologists [26, 27], cross-matched blood [28], and equipment [29] may limit the availability of cancer surgery. Radiation availability has been dynamic in the past decade, but even prior to 2016, when Uganda’s one intermittently functional external beam machine was working 20 + hours per day, it was only meeting 2.6% of the nation’s indicated radiation treatments [30, 31]. A new cobalt-60 radiotherapy machine was inaugurated in January 2018.

Studies are needed to characterize the intervals in the process of obtaining care for cervical cancer as well as to investigate of predictors of delay at each of these intervals in order to identify modifiable barriers and facilitators along the cascade [19, 23]. The purpose of the study was to describe the cascade of cervical cancer care among women ultimately accessing specialty care at government supported tertiary care centers in Kampala, Uganda and to identify barriers and facilitators to timely care across each interval in the care cascade. Understanding which factors contribute to more relative delay during these intervals may help identify ways to reduce overall time to treatment initiation and potentially improve outcomes for women with cervical cancer.

## Methods

The current analysis is part of a larger mixed-methods study which seeks to characterize barriers to cervical cancer care among Ugandan women to inform strategies to minimize delay and improve access to care. Study setting, participant recruitment and data collection activities have been described previously in detail [32]. Women aged 18 or above presenting for care at MNRH and/or UCI with a new diagnosis of cervical cancer were enrolled in the study from May 3, 2017 – September 17, 2018. Patients with pre-cancerous cervical dysplasia (also called cervical intraepithelial neoplasia or CIN) were not included in this study. Data on sociodemographic and reproductive characteristics as well as participants’ care journeys were collected through interviewer-administered surveys conducted in the clinics after initial consultation with a specialist. Data on treatment initiation was captured via phone one to three months later. Medical record abstraction was used to supplement self-reported data.

### Study setting

Mulago National Referral Hospital and UCI are part of the Mulago Hospital Complex, located in Kampala, Uganda, associated with Makerere University College of Health Sciences. They provide comprehensive specialist services for the entire nation. Ninety-nine percent of the national population seeks care through the public sector and access specialty cancer care exclusively at these institutions [33]. Patients without private insurance may present to MNRH through emergency gynecologic triage and may access gynecologic oncology diagnostic and surgical care there. Patients may present directly to UCI’s screening clinic or may be referred for diagnostics, surgery, chemotherapy and/or radiotherapy from the gynecology division of MNRH or from surrounding facilities. Gynecologic oncology specialists and fellowship trainees care for patients at MNRH and UCI through a Memorandum of Understanding between the institutions.

Between March, 2016 and January, 2018, no external beam radiotherapy was available in Uganda; during this time, the Ministry of Health partially subsidized radiation at a private hospital in Nairobi, Kenya, though it is unclear how many patients benefitted from this program. In response to the challenges obtaining radiotherapy 2016–2018, providers at UCI and MNRH developed a protocol for treating locally and regionally-advanced cervical cancer with neoadjuvant chemotherapy followed by possible radical hysterectomy, an evidence-based strategy supported by the American Society of Clinical Oncology (ASCO) Resource-stratified Treatment Guidelines [34–37]. A new cobalt 60 radiotherapy machine became operational at UCI in January, 2018. Brachytherapy was available throughout the study period and may have been recommended for treatment or palliation in conjunction with other therapies. In Uganda, radiotherapy is only available at UCI. There are nominal fees associated with surgery and radiation at MNRH and UCI. The UCI provides chemotherapy free of charge, but drug inventory fluctuates, and stock-outs are common.

### Theoretical orientation

**Fig. 1:**
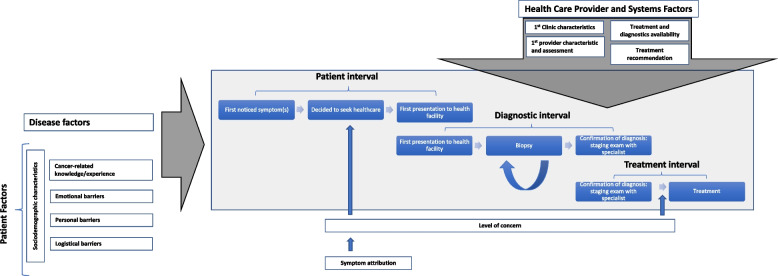
Adapted model of pathways to care—conceptual framework for cervical cancer cascade intervals

Our research was oriented by the Model of Pathways to Care [38, 39], a theoretical model adapted from the Andersen’s Model of Total Patient Delay [40], for understanding the process of seeking and obtaining diagnosis and treatment for cancer. Intervals defined by this model include: patient appraisal and health seeking intervals, characterizing detection of bodily change to perceiving a need to discuss symptoms with a health care provider to first consultation; diagnostic interval, time between first health care consultation to formal diagnosis; and treatment interval, time between diagnosis to treatment initiation. Various contributing factors at multiple levels including the patient, provider, health care system and disease are considered to influence the processes operating within these intervals. The three main levels of factors within this model are ‘patient factors’, representing the individual within their particular social and cultural context (e.g., demographics, previous experience, co‐morbidities, cognitions, and emotions), ‘health care provider and system factors’, representing health care services aspects which can impact patient and provider decisions and behavior (e.g., access to generalist and/or specialist health care including diagnostic procedures and treatment), and ‘disease factors’, the clinical and physiological aspects of the condition (e.g., symptoms and prognosis). Utilizing the intervals specified in this model allows for a standardized, uniform way to report about delay in a patient’s journey to obtaining cancer diagnoses and treatment. Greater precision and transparency in defining this process should, in turn, facilitate the effective design and implementation of interventions to minimize delay. See our conceptual framework (Fig. 1).

### Measures

From patient-reported data on timing of events in their care journey, we calculated three major time intervals in the cervical cancer cascade of care: patient interval, diagnostic interval, and treatment interval. *Patient interval* represents the time (in days) between first noticing symptoms and first presenting to a health care provider, including the process of making the decision to seek care. *Diagnostic interval* represents the time (in days) between first presenting to any health care provider and the consultation with a specialist when diagnosis was confirmed, stage assigned, and treatment recommendations made. *Treatment interval* represented the time (in days) between specialist consultation and initiation of treatment (surgery, chemotherapy, external beam radiation therapy (EBRT) inclusive of radio-sensitizing chemotherapy, or palliative care/hospice). While patients may ultimately receive multi-modal therapy (like chemotherapy followed by hysterectomy or EBRT followed by brachytherapy), the *treatment interval* ends when the first treatment modality started. While brachytherapy was available throughout the study period, this was never the *first* recommended and/or initiated therapy; patients may have accessed brachytherapy later in their care journey, though these data were not captured in this study. Of note, systemic chemotherapy may have been recommended for distant metastatic disease or for non-operable local- or regionally-advanced disease during the time when EBRT was not available in-country.

The survey used in this study was adapted from a validated instrument to measure time intervals and factors correlated with delay in accessing breast cancer care [41]. Where specific dates were unable to be recalled, participants were asked to estimate according to a validated protocol: if participants could not recall exact day, they were asked if the event occurred in the beginning (coded as 5th), middle (coded as 15th) or end (coded as 25th) of the month; if participants could not recall the month exactly, they estimated beginning (coded as February), middle (coded as June) or end (coded at November) or the year [41]. If no estimation could be made, the date was treated as missing.

Patient factors captured included variables representing sociodemographic characteristics, awareness, perceived symptom severity, and perspectives on care access timeline. *Sociodemographic characteristics* captured included age at enrollment (< 40 versus ≥ 40), educational attainment (none, primary, secondary, and other), occupation (farming/domestic work versus industry/business), relationship status (married or partnered versus not). Residence was characterized as rural versus urban (as defined by the Uganda Bureau of Statistics) and distance to specialized care site (MNRH or UCI) calculated (< 15 km, 16–50 km, 51–150 km, ≥ 151 km). *Reproductive history* included parity, HIV status (positive, negative, never been tested, tested but don’t know result), prior cervical cancer screening (yes versus no), current contraceptive use (yes versus no). *Awareness of cervical cancer* was captured through asking whether participants had ever heard of cervical cancer before coming to the doctor (yes versus no), whether they had any friends or family members who had cervical cancer (yes versus no). *Perceived symptom severity* was assessed through asking participants how serious they felt their initial symptom was (not serious, a little serious, moderately serious, serious, very serious), what their level of concern was around this initial symptom (no concern, a little concern, moderate concern, very concerned), and what they initially thought was causing the symptom (cancer, menstrual irregularities, pregnancy, contraception, STI/HIV or other infection, bewitching, other). Patients were also asked to reflect on their own care access timeline, reporting on their impressions of how long it took to decide to seek care, to initially present for care, to see the specialist, and to ultimately start treatment (response options: immediate, quick, a little delayed, and very delayed). Women who reported a little delayed or very delayed were then asked for reasons that contributed to subjective delay (thought symptoms would resolve, didn’t know where to go, scared, embarrassed, sought alternative treatments herbal and/or spiritual in nature, perceived long waits, lack of trust in medical system, and financial constraints).

Health care provider and health systems factors of interest included characteristics of the first clinical encounter: facility type (public versus private), facility level (referral/tertiary, regional versus district/village), provider type (physician, medical officer, nurse or midwife), pelvic exam with speculum to visualize cervix (yes versus no), biopsy (yes versus no); radiation available (yes versus no), number of biopsies done (≥ 2 biopsies, one versus none), cost of biopsy (> 100,000 Ugandan Shillings, ~ $27 USD, versus less), location of biopsy (referral hospital versus elsewhere), number of clinics attended prior to MNRH/UCI (≥ 2 clinics versus one or none), and initial treatment recommendation (chemotherapy, surgery, EBRT, or palliative care).

Disease factors captured included symptomatic (yes versus no), initial symptom (e.g., bleeding, vaginal discharge, pain, other), other symptoms present prior to healthcare presentation (intermenstrual/post-menopausal bleeding, bleeding with sex, vaginal discharge, pain with sex, abdominal/pelvic pain, fatigue). Clinical cancer stage was captured and confirmed by chart abstraction and ultimately classified as early-stage (IA to IIA) versus late-stage (IIB to IVB). Histology (squamous versus adenocarcinoma or adenosquamous) was also abstracted from chart.

### Statistical analyses

Patient characteristics were described with medians with interquartile ranges and proportions. Due to some missing participant self-report data on the exact timing of certain steps in the care cascade (but timing data having been captured on a variety of steps), we implemented an interval censoring approach to our survival analysis [42, 43]. Such an approach accommodates situations where an event of interest is not exactly observed but is known to have occurred within a particular time window. For each interval, we characterized the interval length and estimated a series of proportional hazards regression models using the Weibull survival distribution to explore the impacts of patient, provider and health systems factors, as well as disease characteristics, on interval length. Factors theorized to be associated with the outcomes or those found to be significantly associated in bivariate analysis were considered for multivariate analysis, after ensuring lack of collinearity. In most applications of survival analysis, a longer time to event is preferable due to its interpretation as greater length of time event-free. However, for our characterization of care cascade intervals, a shorter time to event is preferable as our outcomes represent time to completing particular care steps. Hazard ratios (HR) can be interpreted as velocity: the higher the HR, the faster the time to the end of the step. Thus, HRs < 1 indicate slower times to completing a step whereas HRs > 1 indicate faster times to completing a step. Model goodness of fit was established using Akaike’s Information Criteria. All data were analyzed using Stata version 16.0 (Stata Corporation, College Station, TX). *P* values less than 0.05 were considered statistically significant.

### Ethics approval

This study was conducted in accordance with the ethical principles outlined in the Declaration of Helsinki. The study protocol was reviewed and approved by institutional review boards at MNRH (Mulago Research and Ethics Committee, MREC, reference: 1058), UCI (ER-007) and the University of California San Francisco (UCSF) (16-21306). Uganda’s national review board, the Uganda National Council for Science and Technology (UNCST), also reviewed and approved the research (HS29ES). All participants provided written confirmation of informed consent.

## Results

During the study period, there were 268 women ranging in age from 20 to 81 years, with median age 49 (IQR 41–58; Table 1) who sought treatment for cervical cancer at MNRH or the UCI. Over half had primary or higher education (56.3%) and 65.3% worked in farming or domestic work. About one-third (32.1%) lived within 15 km of MNRH or UCI. Most participants had sought care based on symptoms (86.9%) while 13.1% were asymptomatic with cancer detected on screening. Over three-quarters had late-stage cancer on diagnosis (76.1%). The most recommended treatment was chemotherapy (61.2%), followed by EBRT (25.4%), surgery (12.7%), and palliative care (0.8%).

**Table 1:** Sociodemographic and clinical characteristics of women presenting for specialist care for a new diagnosis of cervical cancer, Uganda (*n* = 268)

	All participants	
	*n* = 268	
	n	%
Sociodemographic characteristics		
Age group		
20–29	7	2.6
30–39	47	17.5
40–49	79	29.5
50–59	69	25.7
60–69	39	14.6
≥ 70	16	6.0
Missing	11	4.1
Educational attainment		
None	111	41.4
Primary	100	37.3
Secondary	51	19.0
Other	6	2.2
Occupation		
Farming/domestic work	175	65.3
Industry/business	93	34.7
Marital Status		
Married	121	45.1
Not married	147	54.9
Distance to MNRH or UCI		
< 15 km	86	32.1
16–50 km	37	13.8
51–150 km	76	28.4
≥ 151 km	69	25.7
Residence		
Rural	126	47.0
Urban	142	53.0
HIV Status		
Positive	83	31.7
Negative	179	68.3
Clinical/disease characteristics		
Prior cervical cancer screening		
Yes	68	25.4
No	200	74.6
Symptomatic		
Yes	233	86.9
No	35	13.1
Stage		
IA1	3	1.1
IA2	6	2.2
IB1	26	9.7
IB2	12	4.5
IIA	17	6.3
IIB	96	35.8
IIIA	4	1.5
IIIB	90	33.6
IVA	8	3.0
IVB	6	2.2
Recommended treatment		
Surgery	34	12.7
Chemotherapy	164	61.2
EBRT	68	25.4
Palliative care	2	0.7

*MNRH* Mulago National Referral Hospital, *UCI* Uganda Cancer Institute, *EBRT* External beam radiation therapy

Among the 233 (87%) women who were symptomatic prior to diagnosis, the first symptom noticed for most women was bleeding (65.2%) or abnormal discharge (29.6%; Table 2). A variety of presumed causes of initial symptom were endorsed, most commonly infection (36.5%), abnormal menstruation or menopause (30.5%), versus cancer (10.3%). Over half of women perceived their initial symptoms to be not serious (45.9%) or a little serious (14.6%), and most had no concern (30.9%) or only a little concern (33.9%). Over half of women reported deciding to seek care due to persistent symptoms (53.9%) followed by worsening symptoms (39.7%).

**Table 2:** Initial symptom experiences and interpretation among Ugandan women with symptomatic cervical cancer seeking care at tertiary public facilities (*n* = 233)

	**n**	**%**
Initial symptom identification		
Initial symptom noticed		
Bleeding	152	65.2
Discharge	69	29.6
Other^a^	12	5.2
Initial symptom interpretation		
Presumed cause of initial symptom		
Infection	85	36.5
Abnormal menstruation/menopause	71	30.5
Cancer	24	10.3
Contraceptive side effect	19	8.2
Pregnancy	8	3.4
Other^b^	26	11.2
Perceived seriousness of the problem		
Not serious	107	45.9
A little serious	34	14.6
Moderate	22	9.4
Serious	49	21.0
Very serious	21	9.0
Level of concern about the problem at first		
No concern	72	30.9
A little concern	79	33.9
Moderate	33	14.2
Very concerned	49	21.0
Symptoms present prior to presentation at any healthcare setting		
Abnormal bleeding^c^	163	70.0
Post-coital bleeding	122	52.4
Vaginal discharge	188	80.7
Dyspareunia	51	21.9
Abdominal or pelvic pain	161	69.1
Fatigue	89	38.2

^a^Other symptoms included pain (*n* = 11) and fatigue (*n* = 1)

^b^Other presumed causes included bewitched (*n* = 6), heavy work (*n* = 3), fibroids (*n* = 2), and other medical (*n* = 7)

^c^Intermenstrual or postmenopausal

### Patient interval

Median patient interval was 74 days (IQR 26–238 days). In unadjusted models of patient sociodemographic characteristics (Table 3), no factors were significantly associated with length of patient interval. In multivariate analysis (Table 4), completion of the patient interval was significantly slower those who believed that their symptoms would resolve (aHR 0.37, 95% CI 0.24–0.57), those who reported not knowing where to access care (aHR 0.64, 95% CI 0.47–0.88), as well as those who first sought alternative treatments (including herbal medicine, spiritual healing or other such practices) (aHR 0.70, 95% CI 0.51–0.96). Patient interval completion was significantly faster for those who reported thinking that their initial symptom was serious or very serious (HR 2.14, 95% CI 1.43–3.19), those who were moderately or very concerned about their symptom (HR 2.06, 95% CI 1.45–2.93), and those who believed their first symptom was cancer (HR 1.82, 95% CI 1.12–2.97).

**Table 3:** Sociodemographic factors influencing time to cervical cancer care cascade intervals

	**Patient interval**			**Diagnostic interval**			**Treatment interval**		
	***n* = 233**			***n* = 268**			***n* = 268**		
	**HR**	**95% CI**	**P**	**HR**	**95% CI**	**P**	**HR**	**95% CI**	**P**
Sociodemographic characteristics									
Age < 40	0.82	0.55–1.20	0.305	1.11	0.81–1.54	0.519	1.27	0.88–1.84	0.205
Primary education or higher	1.14	0.84–1.55	0.395	0.78	0.60–1.02	0.070	0.94	0.69–1.28	0.701
Occupation	1.09	0.80–1.48	0.604	1.22	0.93–1.59	0.148	0.77	0.56–1.07	0.117
Married	1.04	0.77–1.41	0.791	1.41	1.09–1.83	0.009	1.40	1.02–1.91	0.032
Parity	1.13	0.83–1.55	0.428	0.97	0.75–1.27	0.842	1.16	0.85–1.58	0.350
Distance to facility ≤ 15 km	1.01	0.73–1.41	0.943	1.89	1.43–2.50	< 0.001	1.47	1.06–2.03	0.020
Urban	0.88	0.65–1.19	0.418	1.11	0.86–1.44	0.424	1.34	0.98–1.82	0.066
HIV positive	0.79	0.57–1.08	0.143	0.98	0.74–1.29	0.865	1.10	0.79–1.52	0.579

**Table 4:** Factors influencing time to cervical cancer care cascade intervals, adjusted models

	**Patient interval**			**Diagnostic interval**			**Treatment interval**		
	***n* = 233**			***n* = 268**			***n* = 268**		
	**HR**	**95% CI**	**P**	**HR**	**95% CI**	**P**	**HR**	**95% CI**	**P**
Patient factors									
Cancer-related knowledge and experience									
Prior screening	0.99	0.67–1.46	0.943	1.27	0.93–1.74	0.136	1.12	0.75–1.65	0.582
Heard of cervical cancer	0.96	0.64–1.44	0.847	0.75	0.53–1.06	0.100	1.04	0.71–1.64	0.825
Know friend w cervical cancer	0.95	0.63–1.43	0.799	0.84	0.60–1.19	0.335	0.79	0.52–1.22	0.292
Symptom perception									
Thought symptom was serious or very serious	2.14	1.43–3.19	< 0.001						
Very concerned about symptom	2.06	1.45–2.93	< 0.001						
Thought 1 st symptom cancer	1.82	1.12–2.97	0.017						
Thought 1 st symptom menstrual	0.83	0.60–1.18	0.315						
Thought symptom would resolve	0.37	0.24–0.57	< 0.001						
Emotional barriers									
Scared	1.20	0.75–1.90	0.443	0.98	0.72–1.33	0.890	0.54	0.23–1.23	0.143
Embarrassed	0.88	0.45–1.71	0.702	0.99	0.67–1.45	0.943			
Doubted provider/recommendation				0.95	0.61–1.47	0.804	NE	-	-
Logistical barriers									
Didn't know where to go	0.64	0.47–0.88	0.007	1.06	0.81–1.38	0.684	1.53	0.89–2.60	0.121
Financial barriers	1.46	0.69–3.06	0.320	0.86	0.63–1.18	0.354	0.93	0.66–1.29	0.650
Anticipated long wait	0.99	0.62–1.59	0.978	0.78	0.57–1.06	0.108	0.68	0.46–1.02	0.062
Needed blood for treatment							0.63	0.37–1.07	0.085
Personal barriers									
Family responsibilities	1.44	0.87–2.37	0.153	1.70	1.17–2.46	0.005	1.30	0.52–3.27	0.578
Difficult to miss work	1.23	0.71–2.14	0.467	1.61	0.98–2.66	0.061	NE	-	-
Pursuing alternative treatment	0.70	0.51–0.96	0.033	0.83	0.63–1.09	0.185	0.69	0.41–1.17	0.168
Disease factors									
Symptoms and clinical characteristics									
Stage IA-IIA (early)	1.17	0.79–1.73	0.427	1.41	1.03–1.95	0.034	1.13	0.77–1.64	0.529
Symptom: bleeding	0.65	0.40–1.04	0.072	0.55	0.35–0.85	0.007	0.75	0.45–1.23	0.248
Symptom: pain	0.77	0.53–1.12	0.171	0.89	0.63–1.26	0.508	1.03	0.68–1.57	0.888
Provider and health systems factors									
First presentation characteristics									
Public				1.27	0.95–1.70	0.104			
MNRH/UCI				2.13	1.20–3.79	0.010			
Physician				0.90	0.59–1.37	0.618			
Speculum				1.07	0.80–1.43	0.657			
Biopsy done				1.23	0.87–1.73	0.244			
Provider suspected cancer				1.13	0.851.51	0.397			
Biopsy characteristics									
≥ 2 biopsies				0.72	0.44–1.18	0.193			
Cost > 100,000 Ug Shillings				1.02	0.73–1.44	0.897			
Biopsy done at MNRH/UCI				1.22	0.92–1.61	0.162			
Clinics attended prior to MNRH/UCI									
≥ 2 Clinics				0.69	0.49–0.97	0.031			
Radiation availability									
Radiation machine operational							0.98	0.70–1.36	0.886
Treatment recommendation									
Chemo							REF	-	-
Surgery							1.23	0.76–1.98	0.394
EBRT							0.89	0.60–1.32	0.566
Palliative care							0.39	0.05–2.85	0.352

All models adjusted for age, education, marital status, parity, and distance to facility

*NE N*ot estimable, *MNRH* Mulago National Referral Hospital, *UCI* Uganda Cancer Institute

Participants with symptoms reflected on the time they took to present for care (Table 5); 27.5% reported accessing care was a little delayed and 28.3% very delayed. Main reasons participants reported for not having sought medical care earlier included thinking symptoms would resolve spontaneously (83.7%), not knowing where to go (57.8%), and seeking alternative treatment (46.4%).

**Table 5:** Patient perspectives on care timeline and reasons for delay along the cervical cancer care cascade

	**Patient interval**		**Diagnostic interval**		**Treatment interval**	
	***n* = 233**		***n* = 268**		***n* = 268**	
	**n**	**%**	**n**	**%**	**n**	**%**
Patient impression of time interval						
Immediate	41	17.6	14	5.2	29	17.4
Quick	62	26.6	56	20.9	65	38.9
A little delayed	64	27.5	83	31.0	45	26.9
Very delayed	66	28.3	115	42.9	28	16.8
Reasons cited for delay						
Thought symptoms would resolve	195	83.7				
Didn't know where to go	133	57.8	145	54.3	24	10.1
Scared^a^	41	17.6	70	26.3	17	7.3
Embarrassed	28	12.0	43	16.2		
Seeking alternative treatment	108	46.4	112	42.3	32	13.4
Financial constraints	9	3.9	187	70.0	153	64.8
Needed blood transfusion for treatment					32	13.4
Too difficult to miss work	31	13.5	30	13.2	1	0.4
Had to care for family	44	19.0	52	19.5	8	3.4
Didn't believe provider/trust tx rec^*b*^			34	12.7	7	2.9
Long wait	51	22.2	88	33.0	54	22.8

^a^For treatment interval, scared of side effects

^b^Didn’t believe the provider or trust in the provider’s treatment recommendation

### Diagnostic interval

Median diagnostic interval was 83 days (IQR 34–229 days). In unadjusted analysis (Table 3), time to diagnostic interval completion was significantly faster for married individuals (HR 1.41, 95% CI 1.09–1.83) and for those living within 15 km of a health facility (HR 1.89, 95% CI 1.43–2.50). Time to diagnostic interval completion was marginally slower for individuals with primary-level education or higher (HR 0.78, 95% CI 0.60–1.02). Among the subset of women under age 40, using family planning was associated with significantly faster time to diagnostic interval completion (HR 3.21, 95% CI 1.13, 9.14; not shown).

In multivariate analysis (Table 4), time to diagnostic interval completion was significantly longer for those who had attended multiple clinics prior to seeking care at MNRH or UCI (aHR 0.69, 95% CI 0.49–0.97), as well as for those who reported symptomatic bleeding (aHR 0.55, 95% CI 0.35–0.85). Time to diagnostic interval completion was paradoxically less for women reporting personal barriers like family responsibilities (aHR 1.70, 95% CI 1.17–2.46). Women whose cancer was early stage (HR 1.41, 95% CI 1.03–1.95) and those who attended MRNH or UCI directly (HR 2.13, 95% CI 1.20–3.79) also had significantly faster diagnostic intervals.

Participants reflected on the time it took from first presenting for any care to receiving their diagnosis and treatment recommendations (Table 5); 31% reported receiving diagnosis was a little delayed and 42.9% very delayed. Most common reasons cited for delay included financial constraints (70%), not knowing where to go (54.3%), and seeking alternative therapies (42.3%).

### Treatment interval

Median treatment interval was 34 days (IQR 18–58 days). In unadjusted modeling (Table 3), time to treatment interval completion was significantly faster for married individuals (HR 1.40, 95% CI 1.02–1.91) and those living within 15 km of a health facility (HR 1.47, 95% CI 1.06–2.03), with a marginal finding for urban residents (HR 1.32, 95% CI 0.98–1.82).

In multivariate analysis, no specific patient, disease or health systems factors were significantly associated with a relatively faster or slower interval completion (Table 4). Notably, no particular treatment modality was associated with a longer or shorter treatment interval. Participants who anticipated a long wait for treatment (HR 0.68, 95% CI 0.46–1.02) and those who required a blood transfusion prior to treatment (HR 0.63, 95% CI 0.37–1.07) experienced marginally longer time to complete the interval.

Participants reflected on the time it took from receiving their diagnosis to initiating treatment (Table 5); 26.9% reported starting treatment was a little delayed and 16.8% very delayed. Main reasons cited for delay were financial constraints (64.8%), long wait time (22.8%), seeking alternative treatment (13.4%) and the need for a blood transfusion prior to starting treatment (13.4%).

## Discussion

We found that, despite a subjective impression of delay, especially during the patient and diagnostic intervals, overall, women with cervical cancer in Uganda were able to present for care, obtain diagnosis and start treatment in a relatively timely fashion. Our conceptual framework allowed us to consider how patient, health system and disease factors either inhibited or facilitated timely completion of different intervals along the cascade to care for women with a new diagnosis of cervical cancer at public tertiary care facilities in Uganda. While we did identify some potentially modifiable barriers in the patient interval (misattribution of symptoms and utilization of traditional care in place of Western medicine), it’s unlikely that interventions to diminish or remove these barriers would significantly impact morbidity or mortality from cervical cancer, given the median duration of the patient interval was less than three months and most patients had experienced hallmark symptoms of advanced disease before even attempting to seek care.

Similar to 20 year-old estimates from the Kampala Cancer Registry [44], as well as to regional estimates [19, 45–50], approximately three-fourths of participants in our study had late-stage disease at diagnosis. The lack of change, while disappointing, is not surprising as there has been little expansion of screening and/or vaccination services nationwide during the last few decades. Vaccination and screening are the strongest strategies to decrease incidence, morbidity and mortality of cervical cancer [51–54]. Immediate rapid scale-up of primary and secondary prevention programs (80–100% global vaccine coverage and 70% HPV-based screening coverage) would be required to meet the WHO Director-General’s global call for action to eliminate cervical cancer in the next century [55].

In this study, the patient interval, median duration 74 days, was delayed among participants who incorrectly believed their symptoms would resolve on their own (i.e., without medical care). Misattribution of symptoms was also common; nearly 90% of participants did not suspect cancer. Similarly, qualitative studies from Northern Uganda have showed that incorrect attribution and minimization of symptoms were common and were hypothesized to delay presentation to care [21–23].

Our findings suggest that engaging in alternative therapies also significantly prolonged the patient interval and, while not significantly associated with relatively slower completion of diagnostic or treatment intervals, participants did cite “seeking alternative treatment” as a common qualitative reason for delay during these intervals. Qualitative data from Northern Uganda suggests that traditional medicines are commonly used for treatment of cervical cancer and often thought to be superior to Western medical therapies. This same study reported community perceptions of cervical cancer care in hospitals to be congested, confusing and disrespectful, contributing to a distrust of Western medical treatments for cervical cancer [56].

During the diagnostic interval, we found that early-stage disease was associated with a faster journey through the health system to obtain a diagnosis, whereas a study from Northern Uganda, a more rural setting, did not find a significant association between stage and delay (defined as > three months from symptom perception to diagnosis) [19]. We also found that direct presentation to tertiary care center (first presentation) was associated with a faster diagnostic interval. There are likely several unmeasured factors that contribute to presenting earlier in the disease course and directly to an urban tertiary care center. Overall, it is notable that the median diagnostic interval in our study, 83 days, is below that NBCCEDP quality standard of ≤ 90 days for diagnostic interval [15].

Our absolute median duration of the treatment interval, 39 days from diagnosis to starting treatment, was similar to a reported duration at a specialty center in Botswana [57]. Whereas other studies from the region have reported median duration > 100 days [48, 58], substantially longer than our reported treatment interval (though our reported treatment interval is likely a conservative estimate). Both the specialty center in Botswana and MNRH/UCI have regular multidisciplinary Tumor Board discussions, perhaps improving communication between the various subspecialties providing care for these patients. Disappointingly, neither early-stage disease (better chance of survival/cure with appropriate treatment) nor a recommendation for curative hysterectomy was associated with a shorter treatment interval. As discussed previously, data from high-income countries, where cervical cancer is more likely be diagnosed at an earlier/highly curable stage, has associated treatment interval delay to worse outcomes [17, 18]; however, the median duration of the treatment interval in our study, 39 days, is well-below the 3-month threshold defining delay in these studies, again calling into question whether there is room for improvement.

### Strengths and limitations

This is the first study to examine relative barriers and facilitators to care at specific intervals in the cervical cancer care cascade in Uganda. Our conceptual framework (Fig. 1) is based on the Model of Pathways to Treatment [38], a theoretical approach that informs measurement and description of delay in accessing cancer care in a generalizable way across cancer sites, potentially allowing for greater consistency in reporting delay and allowing for better comparison of delay across studies in order to advance existing knowledge and effectively design interventions. Because the process of obtaining care for cancer is heterogeneous, the granular interval-specific data allow us to consider the role for specific interventions to address delay at various points on the care cascade.

A major limitation of this study is that recall bias could have affected the precision of measurement of intervals and other variables. We have tried to limit recall bias by using validated measures with demonstrated reliability for estimation of these intervals [41], and through the interval-censoring analytic approach. Another limitation is limited generalization of our findings given that the study population was a convenience sample of women who were able to access specialized cancer care at national referral centers. Three-fourths of women in this study, who were able to navigate to a public tertiary care facility, had advanced disease. While this is possibly an underestimate of proportion at large with late-stage disease, it is also possible that women with very early-stage disease were treated locally with simple hysterectomy. The particular delays experienced by women in this study may not be generalizable to the national population of women with cervical cancer, especially those experiencing such significant delay that they never made it to diagnosis or treatment.

### Meaningful implications

Our findings suggest that interventions that aimed at improving identification of cervical cancer symptoms, perhaps undertaken in conjunction with traditional healers, may expedite the patient interval and, thus, presentation to care. Misattribution of symptoms was common and was associated with delay in presenting for care. While most women in the present study reported general awareness of cervical cancer [32], perhaps there is a role for education efforts specifically regarding signs and symptoms of cervical cancer, especially as those women who did suspect their symptoms were serious and/or due to cancer had a relatively faster patient interval. A similar vulnerability (knowledge) has been associated with delay among women with endometrial cancer in the United States and highlighted as a potential area for evidence-based intervention [59, 60].

Acknowledging that many participants are not only using alternative therapies including traditional medicine and spiritual healing, but citing utilization as a reason for delay, perhaps re-framing these modalities as complementary, rather than alternative, with engagement and buy-in from traditional practitioners, could facilitate earlier and potentially more streamlined referral to Western medical care for cervical cancer. In Uganda, traditional healers are highly respected authoritative figures and commonly consulted, especially by women, often prior to or instead of engagement with allopathic clinics. There is an estimated one traditional healer for every 200–400 people [61], while a level II health center (providing basic outpatient medical services, staffed by nurses) serves a catchment area of 5000 and just 71% of Ugandans live within a one-hour walk of such a facility [62]. There are examples of effective collaborations among traditional allopathic medicine in Uganda, especially in the realm of HIV and family planning care [63]; but there is likely a potential for greater collaboration as evidence suggests mutual mistrust and competition between traditional healers and Western medical practitioners [64].

Interventions designed to streamline referrals to national referral centers (MNRH and UCI, the only public facilities able to provide cancer care) may hasten the diagnostic interval. We did find that patients who went to two or more clinics prior to MNRH or UCI experienced longer diagnostic intervals, while those who presented directly to the national referral centers completed this interval quicker. However, a practical solution to streamline timely referrals is challenging. The health system in Uganda operates on a decentralized referral system with government-trained Village Health Team (VHT) as intended first point of contact with subsequent referral based on health needs, but great disparities exist between rural and urban populations with the most vulnerable poor rural Ugandans facing disproportionately worse access to even basic health centers [62, 65]. Given an under-funded health sector with inequitable distribution [33], an effective intervention would likely require significant restructuring of the health sector. Alternatively, disseminating specialty cancer care beyond the urban national referral centers, a model undertaken by the Rwandan Ministry of Health in their creation of the Butaro Cancer Center of Excellence (BCCOE) [46], may improve timeliness to diagnosis and potentially treatment.

The anticipated long wait times, which seemed to lengthen the treatment interval, implies an unmet need for treatment, be it a radical hysterectomy, primary radiation and/or chemotherapy. Worldwide, the burden of cancer morbidity and mortality disproportionately affects LMICs (78% of global years of life lost and 77% of disability-adjusted life years due to cancer), yet less-developed countries account for just 6% of total resources spent on cancer care globally [66, 67]. Amidst the global COVID-19 pandemic, now more than ever before, the international community, must recognize the inter-connected nature of our health systems and re-calibrate it’s response to widespread inequity to enable sufficient funding for capacity building and treatment. Part of the WHO Director-General’s call for global elimination of cervical cancer is for 90% of women with invasive cancer to access effective treatment, a target not achieved in this cohort [32].

### Unanswered questions

While our findings suggest interval-specific barriers and facilitators to timely completion of the cervical cancer care cascade, given that most participants had symptoms motivating presentation to care (87%) and that most were advanced stage at diagnosis (76%), interventions aimed at decreasing the median duration of intervals, may not result in substantial reduction in morbidity or mortality from cervical cancer. Again, vaccination and screening are the most effective strategies to decrease morbidity and mortality from cervical cancer and must be a primary and immediate focus for intervention [54].

Expanding access to screening may result in increasing the proportion of cervical cancer diagnosed at an early stage, when curative hysterectomy is possible. World-wide, early-stage cervical cancer is often asymptomatic and incidentally diagnosed during routine screening. Cure rates are highest for early-stage cervical cancer. While timely access to treatment is certainly important for palliation of advanced symptoms, like pain and bleeding, hastening treatment for advanced disease by a matter of days to even weeks will not likely decrease morbidity or mortality. A cohort study in the United States recently found that while median time to initiate definitive chemoradiation for locoregionally advanced cervical cancer increased from 36 to 44 days between 2004–2014, survival was not impacted [68].

Efforts to expand primary and secondary prevention are of utmost importance for women in Uganda as well as world-wide. On the other side of the spectrum, hospice and palliative care, though widely available in Uganda and subsidized, seem to be poorly utilized. Given the proportion of women with late-stage, less-likely curable cervical cancer, coupled with the significant portion of participants who had not yet accessed treatment during the study period, future research is needed to understand the specific barriers among patients and providers to uptake and referral to palliative care and hospice.

## Conclusions

We identified potentially modifiable barriers along the cervical cancer care cascade including: misattribution of symptoms, preferential use of local healers, lack of streamlined referral and anticipated long wait times. Interventions targeting these factors may improve care timeliness but are unlikely to significantly improve morbidity or mortality given the average stage at symptomatic presentation. While expanding cervical cancer screening and vaccination are the most effective strategies to improve morbidity and/or mortality, timely care is important for palliation of symptoms of advanced disease.

## Data Availability

The datasets generated by the survey research during and/or analyzed during the current study are available in the Dryad repository (doi:10.5061/dryad.dv41ns24m): [https://datadryad.org/stash/share/upLAOiCWeBLV8Bu4Zdee67d8Df5rfQmF9pdeqAgCLvw](https:/urldefense.com/v3/__https:/datadryad.org/stash/share/upLAOiCWeBLV8Bu4Zdee67d8Df5rfQmF9pdeqAgCLvw__;!!LQC6Cpwp!o9YXnVa9_eyl49OC0E2Dwdp2JkpQMxwJ1pkyPhxq2b3wVgVYw5hBhxgnZaGSLMd2bDd-pr9rt-zdifKon1TDJffsshv7DA24kA$).

## Abbreviations

IQR: Interquartile range
HR: Hazard ratio
aHR: Adjusted hazard ratio
CI: Confidence interval
MNRH: Mulago National Referral Hospital
UCI: Uganda Cancer Institute
LMICs: Low- and middle-income countries
NBCCEDP: National Breast and Cervical Cancer Early Detection Program
EBRT: External beam radiation therapy
STI: Sexually transmitted infection
HIV: Human immunodeficiency virus
MREC: Mulago Research and Ethics Committee
UCSF: University of California San Francisco
UNCST: Uganda National Council for Science and Technology
